# A Novel Transfer Function Based Ring-Down Suppression System for PMUTs

**DOI:** 10.3390/s21196414

**Published:** 2021-09-25

**Authors:** Zhipeng Wu, Wenjuan Liu, Zhihao Tong, Songsong Zhang, Yuandong Gu, Guoqiang Wu, Alexander Tovstopyat, Chengliang Sun, Liang Lou

**Affiliations:** 1 The Institute of Technological Sciences, Wuhan University, Wuhan 430072, China; whuwuzhipeng@whu.edu.cn (Z.W.); lwjwhu@whu.edu.cn (W.L.); wuguoqiang@whu.edu.cn (G.W.); alxtov@whu.edu.cn (A.T.); 2 The Shanghai Industrial μTechnology Research Institute, Shanghai 201899, China; zhihaotong0929@163.com (Z.T.); songsong.zhang@sitrigroup.com (S.Z.); alex.gu@sitrigroup.com (Y.G.)

**Keywords:** blind area, PMUTs, ring-down vibration suppress, transfer function

## Abstract

In this paper, a novel ring-down suppression system based on transfer function is proposed for the first time to suppress the ring-down time and decrease the blind area of PMUTs (Piezoelectric Micromachined Ultrasonic Transducers). This suppression system includes a transfer function and a simple P (proportion) controller, which can reduce the ring-down time without degrading any performances of PMUTs. The transfer function serves as a virtual PMUT device, feeding its output into the P controller; then, the P controller generates a suppression signal to the actual PMUT device. The ring-down time of a 115-kHz PMUT array is demonstrated to be reduced by up to 93% through the suppression system. In addition, the P controller has been experimentally optimized, reducing the blind area of the PMUT array by about 40%. Moreover, a low ring-down PMUTs system design guideline is established, which is practical and straightforward for industrial scenarios. Finally, the system can be easily integrated into ASIC (Application Specific Integrated Circuit).

## 1. Introduction

Ultrasonic transducers have been widely used for rangefinders, especially at short (<10 m) distances, since the relatively low speed of sound alleviates the high-speed electronics requirements [1,2,3]. PMUTs (Piezoelectric Micromachined Ultrasonic Transducers) are particularly attractive in applications requiring multiple transducers for range finding due to their small size and low power consumption. Besides, PMUTs work without DC bias and can be compatible with the complementary metal-oxide-semiconductor (CMOS) fabrication process.

**Figure 1:**
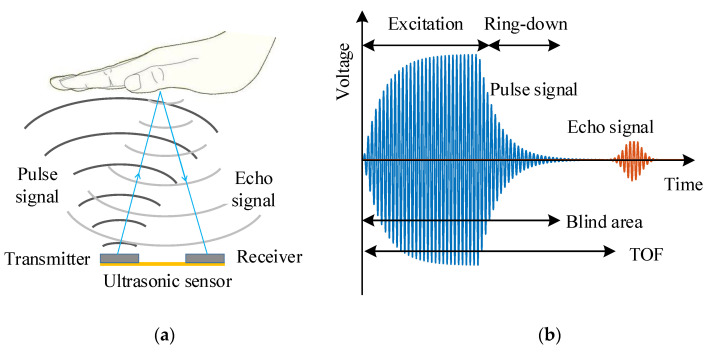
Illustration of (**a**) pulse-echo mode and (**b**) the cause of blind area.

When the PMUTs based ultrasonic sensor works in pulse-echo mode, the transmitter generates the pulse signal, and the echo signal is caught by the receiver, as shown in Figure 1a. Then the distance can be obtained through the difference between the arrival time of the echo signal and the emission time of the pulse signal (Time of flight, TOF). When the excitation signal is removed, the PMUTs continue to vibrate due to mechanical inertia called the ring-down vibration or “tailing” [4], as shown in Figure 1b. During the ring-down stage, the echo signal will be affected by the ring-down vibration of the PMUTs, such as overlapped or distortion, which fails to distinguish the echo signal and TOF [4,5]. In pulse-echo mode, the first echo signal that can be received must arrive later than the end of pulse signal transmission. The duration of the pulse signal is the blind area for PMUTs. The ring-down vibration is the main cause of the blind area for PMUTs. The PMUTs generally have lower bandwidth and higher quality factor (*Q*) than bulk piezoelectric transducers and Capacitance Micromachined Ultrasonic Transducers (CMUTs), which results in longer ring-down time and more unsatisfactory performance in practical applications [5,6,7,8,9,10,11]. Suppression of ring-down vibration can reduce the blind area and improve the ranging distance of the PMUTs.

At present, many efforts have been made to reduce the ring-down time or blind area of ultrasonic transducers, such as full-bridge acoustic emission [4], parallel resistors [12,13], Golay complementary pairs [14], amplitude detection [15], etc. The above methods are usually verified using bulk piezoelectric transducers, not PMUTs with much smaller bandwidth and higher *Q*. Yao et al. [4] proposed a full bridge acoustic emission technique based on bootstrap gate driver (BGD) and metal-oxide-semiconductor field-effect transistor (MOSFET). When the excitation signal is removed, the full-bridge acoustic emission technique quickly consumes the residual energy. Chang et al. [12,13] suggested a parallel resistor to reduce DC time constant and DC response time of the transient response, induced immediately after an AC voltage connected to a bolt-clamped Langevin transducer (BLT). The above methods [4,12,13] are effective by increasing the complexity of the peripheral circuit. Hernández et al. [14] presented an encoding technique based on Golay complementary pairs, where the dimension of the blind zone is reduced to negligible distances using specially designed features from the binary sequences. The encoding technique is not suitable for PMUTs due to its small bandwidth. Few methods have been proposed to reduce the ring-down of PMUTs and can be generally divided into two categories: suppression system design [16,17,18] and device structure modification [19,20]. Liu et al. [16,17] proposed a phase shift method to reduce the ring-down of PMUTs, and the optimized ring-down time accounts for less than 15.1% of the natural ring-down time in the air. Pala et al. [18] proposed optimizing the duration of the inverse signal in the phase-shift cancellation scheme. The results show that the ring-down time is shortened by 13% and 7.5% for a single element and the 80-element array, respectively. Kusano et al. [19,20] reported the effects using DC-biased lead-zirconate-titanate (PZT) PMUTs, in which the polarization and stress are controlled accordingly. At the optimal bias voltage, the (1,1) mode and (1,3) mode nearly overlap, increasing the bandwidth by a factor of eight and decreasing the ring-down time by about 82% relative to the zero DC-biased state. Modifying the device structure will change characteristics of PMUTs, and usually involves much fabrication-related work. Suppression system design will not affect the PMUTs design and their fabrication. It is worth noting that the existing suppression systems are sensitive to system parameters.

To suppress ring-down time and decrease blind area of PMUTs, a novel ring-down suppression system based on PMUTs’ transfer function with a simple P (proportion) controller is proposed in this paper. The transfer function serves as a virtual PMUT device, feeding its output into the P controller; then, the P controller will generate a suppression signal to the actual PMUT device. The suppression system only works on the ring-down stage, which will not impact the performances of PMUTs. Moreover, this approach proves a significant suppression effect and is insensitive to system parameters. A 93% ring-down time reduction is demonstrated experimentally using a 115-kHz PMUT array, and its blind area is reduced by about 40% after optimizing the P controller. Compared with the prior art, this suppression system provides a common solution, proving good stability and straightforward operation with minimum complexity of peripheral circuits. Moreover, design guidelines have been extracted for the suppression system as well. This work demonstrates an effective method of reducing blind area and improving ranging distance, which can be applied to a wide spectrum of applications, including short distance rangefinder, gesture identification, ultrasonic proximity sensing skin, etc.

## 2. Structure and Characterizations of PMUTs

**Figure 2:**
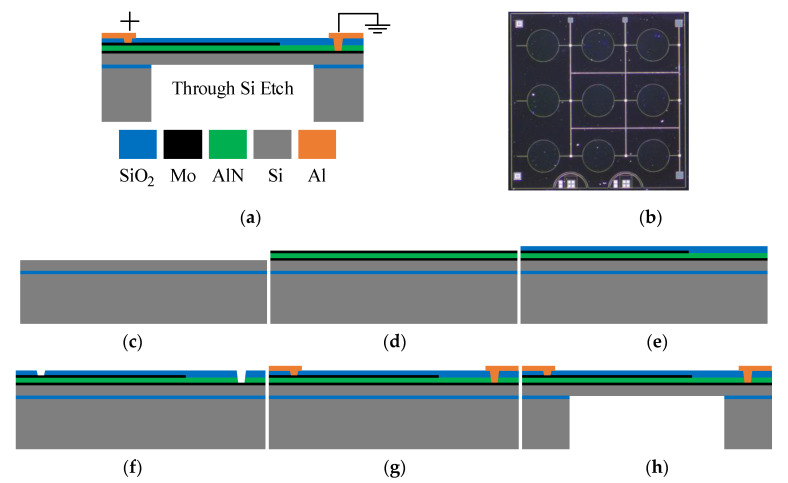
The PMUT array: (**a**) cross-sectional view, (**b**) optical microscopic image, (**c–h**) process flow.

The PMUTs are fabricated on SOI (Silicon on Insulator) wafer. As shown in Figure 2a, the PMUTs are comprised of a thin film Aluminum Nitride (AlN) piezoelectric layer sandwiched between two molybdenum (Mo) electrodes and a Silicon (Si) passive layer. The corresponding geometric parameters of the PMUTs are summarized in Table 1. As shown by the optical microscopic image (Figure 2b), the PMUT array comprises 9 (3 × 3) independent PMUTs. Because of the piezoelectric effect of AlN, this structure can transform electrical energy into acoustic energy and vice versa. Specifically, when applying an appropriate AC signal to the top and bottom electrodes, the transverse internal stress produced by the AlN layer will make the diaphragm vibrate and generate ultrasonic waves. Conversely, the electrodes will detect electronic signals when ultrasonic waves hit the diaphragm.

The process flow of the PMUT array is shown in Figure 2c–h [21]. (c) Customize a single-side polishing SOI wafer. (d) Multiple layers of the bottom Mo electrode, AlN layer, and top Mo electrode are sputtered on the SOI wafer. (e) The top Mo electrode is patterned by plasma etching, and an oxide layer is deposited to form the isolation layer. (f) The etching of SiO_2_ and AlN follows it to pattern the via opening for the top and bottom electrodes. (g) The Aluminum (Al) leads and bonding pads are subsequently deposited and patterned. (h) The deep reactive ion etching (DRIE) is conducted from the backside of the SOI wafer to release the patterned membrane. The whole process is completed at Shanghai Industrial μTechnology Research Institute (SITRI).

**Figure 3:**
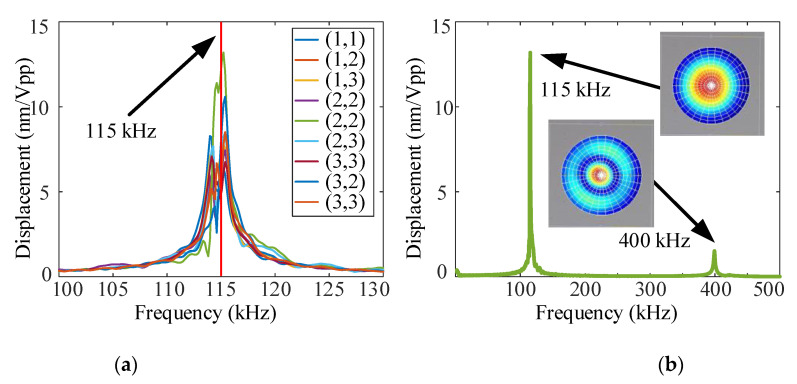
The resonant frequency and mode shape of (**a**) 9 independent PMUTs and (**b**) the central element in the PMUT array.

The resonant frequency and mode shape are characterized using a Laser Doppler Vibrometer (LDV, Polytec UHF-120). The measurement results of 9 independent PMUTs in the PMUT array are shown in Figure 3. The multilayer structure of PMUTs inevitably suffers from frequency variation, which is highly processed dependent. As shown in Figure 3a, a change in the resonant frequency is clearly observed. Although there is a frequency variation in the PMUT array, the bandwidth of all elements overlaps, mostly. Moreover, the resonance frequency of the central element can be used to represent the resonance frequency of the PMUT array. According to the measurement results, the 1st resonance frequency of the central element is about 115kHz; the −3dB bandwidth is 0.908kHz, the *Q* is 126.65, and the displacement sensitivity of the central point is 13.2 nm/V_pp_.

## 3. Design of The Suppression System

### 3.1. Transfer Function of PMUTs

**Figure 4:**
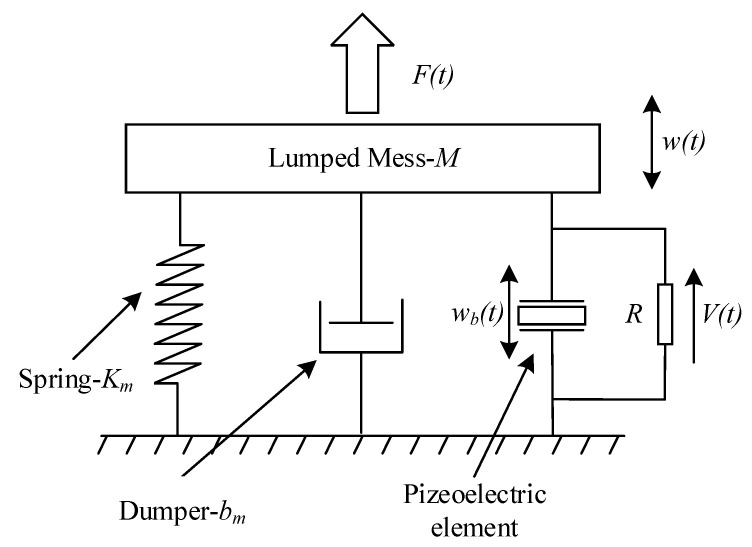
The spring-mass-damper-piezo model of PMUTs.

The PMUTs are a spring-mass-damper-piezo model, as shown in Figure 4 [16,22,23,24,25]. In this model, the PMUTs consist of a mass with lumped mass *M*, a damper with a damping coefficient of *b_m_*, a spring with a spring coefficient of *K_m_*, and a piezoelectric element. *w_b_*(*t*) is the displacement due to the piezoelectric effect, and *w*(*t*) is the displacement output by the model. The piezoelectric element is connected to the drive circuit, and a load *R* is directly connected to the piezoelectric element. The voltage at the ends is *V*(*t*). The dynamic equation of the above model is:

(1) $$M\ddot{w}(t)+b_{m}\dot{w}(t)+K_{m}w(t)+\Theta_{bc}V(t)=M{\ddot{w}}_{b}(t)$$

where Θ*_bc_V*(*t*) denotes the piezoelectric coupling force generated by voltage *V*(*t*), Θ*_bc_* denotes the backward coupling constant. According to the *e*-type piezoelectric equation, the relationship between *V*(*t*) and *w_b_*(*t*) can be expressed as:

(2) $$C_{pe}\dot{V}(t)+\frac{V(t)}{R}-\Theta_{fc}{\dot{w}}_{b}(t)=0$$

where *C_pe_* denotes the effective capacitance between the top and bottom electrodes, Θ*_fc_* denotes the forward coupling constant.

One could obtain Equations (3) and (4) by performing Laplace transform on Equations (1) and (2), respectively,

(3) $$w(s)(Ms^{2}+b_{m}s+K_{m})+\Theta_{bc}V(s)=w_{b}(s)Ms^{2}$$

(4) $$C_{pe}sV(s)+\frac{V(s)}{R}-\Theta_{fc}sw_{b}(s)=0$$

Then the transfer function of the excitation signal to the displacement of PMUTs can be expressed as:

(5) $$H(s)=\frac{\frac{MC_{pe}}{\Theta_{fc}}s^{2}+\frac{M}{\Theta_{fc}R}s+\Theta_{bc}}{Ms^{2}+b_{m}s+K_{m}}$$

It could be seen from Equation (5), the PMUTs are a second-order system with 2-zeros and 2-poles.

### 3.2. The Theory of the Suppression System

One could obtain the Dynamic equation of PMUTs through Equations (1) and (2).

(6) $$\ddot{w}(t)+\frac{b_{m}}{M}\dot{w}(t)+\frac{K_{m}}{M}w(t)=\frac{C_{pe}}{\Theta_{fc}}\ddot{V}(t)+\frac{1}{\Theta_{fc}R}\dot{V}(t)-\frac{\Theta_{bc}}{M}V(t)$$

When the excitation signal is removed, the PMUTs are in the free vibration stage and *V*(*t*) = 0. Then, Equation (6) can be converted to:

(7) $$\ddot{w}(t)+\frac{b_{m}}{M}\dot{w}(t)+\frac{K_{m}}{M}w(t)=0$$

The solution of Equation (7) is:

(8) $$w=A_{0}e^{-\beta_{e}t}\cos(\sqrt{\omega_{0}^{2}-\beta_{e}^{2}}t+\varphi_{0})$$

where *A_0_* denotes the initial displacement of the PMUTs, $\omega_{0}^{2}=K_{m}/M$, $2\beta_{e}=b_{m}/M$.

When the excitation signal is removed, the PMUTs are excited by an additional excitation signal. The same cosine signal as the free vibration stage with a negative proportional parameter is used as the additional excitation signal, which is set as $V(t)=-V_{0}\cos\left(\omega_{e}t+\varphi_{v}\right)$. Then, Equation (6) can be expressed as:

(9) $$\ddot{w}(t)+\frac{b_{m}}{M}\dot{w}(t)+\frac{K_{m}}{M}w(t)=-F_{0}\cos(\omega_{e}t+\varphi )$$

where

$$F_{0}=\sqrt{A_{V}^{2}+B_{V}^{2}},\;\varphi =\varphi_{V}+\arctan\frac{A_{V}}{B_{V}}+\frac{\pi }{2}$$

$$A_{V}=\frac{C_{pe}\omega_{e}^{2}V_{0}}{\Theta_{fc}}+\frac{\Theta_{bc}}{M}V_{0},\;B_{V}=\frac{\omega_{e}V_{0}}{\Theta_{fc}R}$$

The solution of Equation (9) is:

(10) $$w=A_{0}e^{-\beta_{e}t}\cos(\sqrt{\omega_{0}^{2}-\beta_{e}^{2}}t+\varphi_{0})-A_{F}\cos(\omega_{e}t+\varphi )$$

where

$$A_{F}=\frac{F_{0}}{M\sqrt{{\left(\omega_{0}^{2}-\omega_{e}^{2}\right)}^{2}+4\beta_{e}^{2}\omega_{e}^{2}}},\tan\varphi_{0}=\frac{2\beta_{e}\omega_{e}}{\omega_{0}^{2}-\omega_{e}^{2}}$$

It could be seen from Equation (10), $A_{0}e^{-\beta_{e}t}\cos(\sqrt{\omega_{0}^{2}-\beta_{e}^{2}}t+\varphi_{0})$ is the free vibration displacement of the PMUTs when the excitation signal is removed and $-A_{F}\cos(\omega_{e}t+\varphi )$ is the additional attenuation term of the PMUTs displacement. The additional excitation signal generates the additional attenuation term. The above analysis shows that the suppression signal is generated by the reference signal with a specific gain for the ring-down vibration.

### 3.3. The Design of the Suppression System

**Figure 5:**
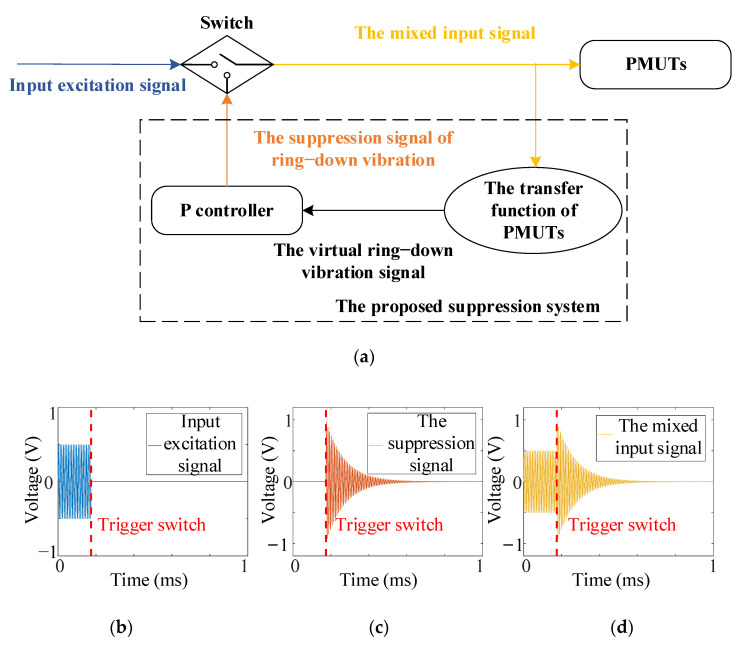
The suppression system based on the transfer function of PMUTs: (**a**) system architecture, (**b**) the input excitation signal, (**c**) the suppression signal of ring-down vibration, (**d**) the mixed input signal.

According to the above theory, the ring-down signal can be used as a reference signal, and the ring-down vibration of PMUTs can be reduced through the reference signal with a specific gain. Then the suppression system based on the transfer function of PMUTs is designed, illustrated in Figure 5. The suppression system consists of the transfer function of PMUTs and a P controller. The transfer function of PMUTs serves as a virtual PMUT device and generates the virtual ring-down vibration signal. Since the object value of the controller is 0, the input error signal of the P controller is the same as the virtual ring-down vibration signal. Then the P controller outputs the suppression signal of ring-down vibration to the actual PMUT device by multiplying the virtual ring-down vibration signal through the proportional parameter. The switch isolates the input excitation signal and the suppression signal of ring-down vibration to avoid the influence of the suppression system on the echo signal. The mixed input signal finally applied to the actual PMUT device is divided into the input excitation signal and the suppression signal through the switch. Moreover, the proposed system suppresses the ring-down time of PMUTs in a controlled manner and has no impact on the performances of PMUTs.

Due to the high resonance frequency of PMUTs, the hardware requirements to implement the real-time system is high if the ring-down signal of real PMUTs is used to feedback into the P controller. Therefore, the transfer function of PMUTs is used as a model to predict the vibration of PMUTs. The transfer function of PMUTs is obtained through the system identification in the toolbox of MATLAB since the transfer function of PMUTs obtained from Equation (5) is very difficult. The controller is the core to generate the suppression signal of ring-down vibration. Since the theory in Section 3.2. can be implemented through a basic P controller, the P controller is chosen as a start to verify the proposed suppression system. The proportional parameter of the P controller depends on the actual scenarios, which can control the ring-down time or blind area of PMUTs. The suppression signal is calculated through MATLAB, and it always attenuates with time. Thus, the mixed input signal provides limited energy. In the meantime, the PMUTs are passive devices. In such a case, the stability of the P controller-based system can be guaranteed.

Moreover, a low ring-down PMUTs system design guideline is also proposed based on the suppression system. Firstly, the transfer function is obtained through the measurement results in the actual environment. Then, the suppression system is designed and attached to the PMUTs based sensors. The system does not need many calculation resources and is easy to integrate into ASIC (Application Specific Integrated Circuit). In addition, since the influence of actual environmental conditions on the PMUTs is included in the transfer function, the proposed suppression system has the ability to accommodate the changing environment.

## 4. Results and Analysis

Experiments are conducted to verify the performances of the suppression system. The 115-kHz 3 × 3 PMUT array shown in Figure 2b is utilized as an experimental device. Firstly, the vibration signal of the PMUT array is obtained through LDV. Moreover, the transfer function of the PMUT array can be gutted through system identification. Then, the suppression system is implemented in MATLAB/Simulink, while the suppression signal of ring-down vibration can be generated simultaneously. Finally, the mixed input signal is used to excite the PMUT array through a waveform generator to verify the effectiveness of the suppression system.

### 4.1. Experimental Setup

**Figure 6:**
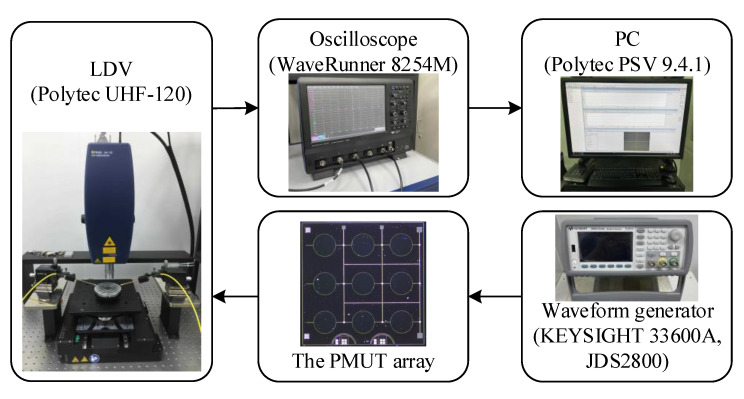
Functional diagram of the LDV setup for vibration displacement.

The vibration displacement of the PMUT array is characterized using LDV. The measurement setup is shown in Figure 6. The excitation signals are generated using a waveform generator (KEYSIGHT 33600A or JDS2800). The raw signal from the photodetector is collected with an oscilloscope (WaveRunner 8254M). Furthermore, the in-phase quadrature (IQ) vibrational data is demodulated, and the mode shape is presented on a controlling personal computer (PC) using Polytec PSV 9.4.1 software.

**Figure 7:**
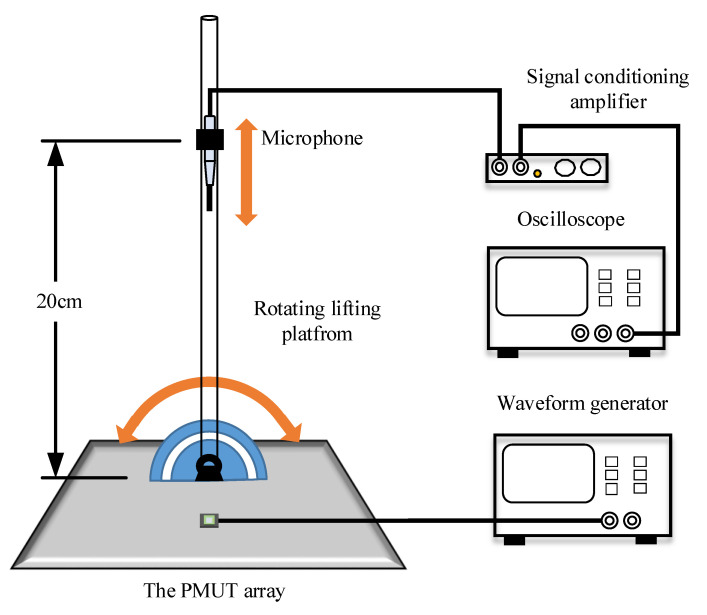
Schematic of microphone experimental setup.

The acoustic characteristics are measured by a microphone, as shown in Figure 7. The PMUT array is placed at the center of the rotary lifting platform. During measurement, the PMUT array is excited by a waveform generator. Ultrasound wave generated by the PMUT array is measured using a microphone (B&K Type 4138-L-006) connected to a signal conditioning amplifier (B&K Type 1708). The microphone is placed in a metal clamp 20 cm away from the devices. Moreover, the corresponding output signal is recorded and displayed by an oscilloscope (KEYSIGHT DSOX2014A).

### 4.2. Experiment of the Transfer Function

The system identification results are shown in Table 2 through the input excitation signal and the vibration signals of the central point of the central element in the PMUT array. The input excitation signal is a 115-kHz sine signal of 20 cycles with an amplitude of 1V_pp_. The transfer function of the PMUT array is obtained by the 0-zero and 2-pole second-order system (0-2) model, the 1-2 model, the 2-2 model, the 0-3 model, the 1-3 model, the 2-3 model, the 3-3 model respectively. The FED (Fit to estimation data) is the degree of fit between the objective transfer function and the result of the system identification. The 2-2 model has the best FED, consistent with the above theory in Section 3. The FED of the PMUT array cannot reach a high value since the inevitably a mutual influence between the elements of the PMUT array [26]. This nonlinear influence cannot be described by the linear transfer function of the PMUT array. The FED can be improved if the experimental device is a single PMUT. In practical application, the PMUTs are always an array due to the inferior output of a single element. At the same time, the FED of the transfer function based on the 2-2 model is also high (>80%) enough to describe the characterize the vibration of PMUTs. Therefore, the transfer function based on the 2-2 model is used for the suppression system.

### 4.3. Verification of the Suppression System on Reducing the Ring-Down Time and Blind Area

**Figure 8:**
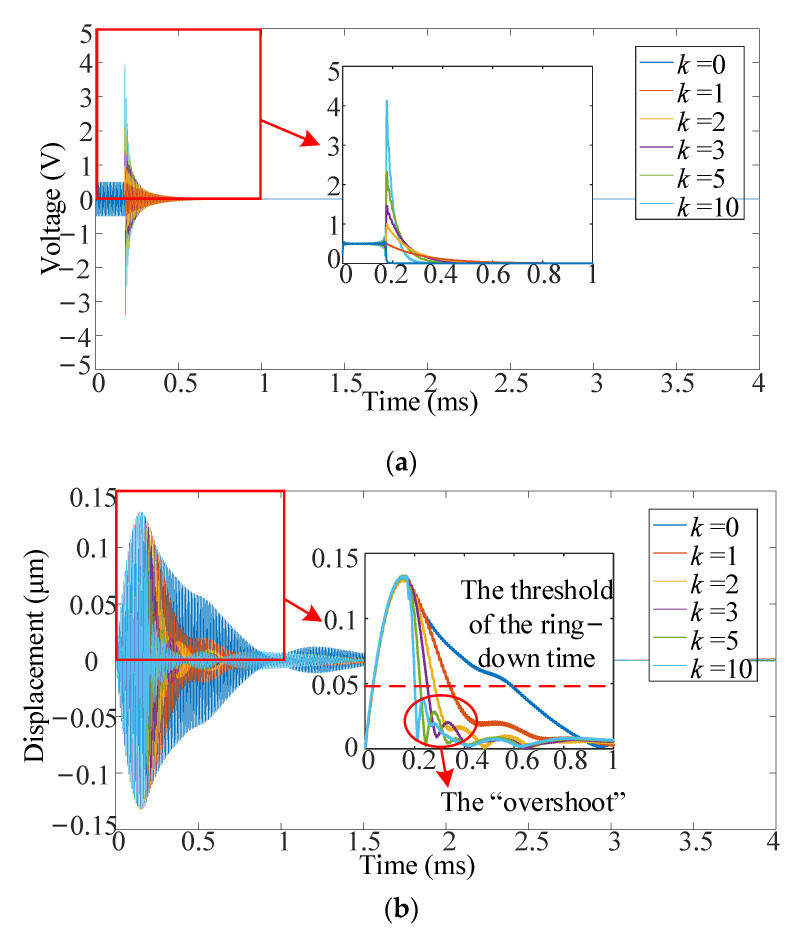

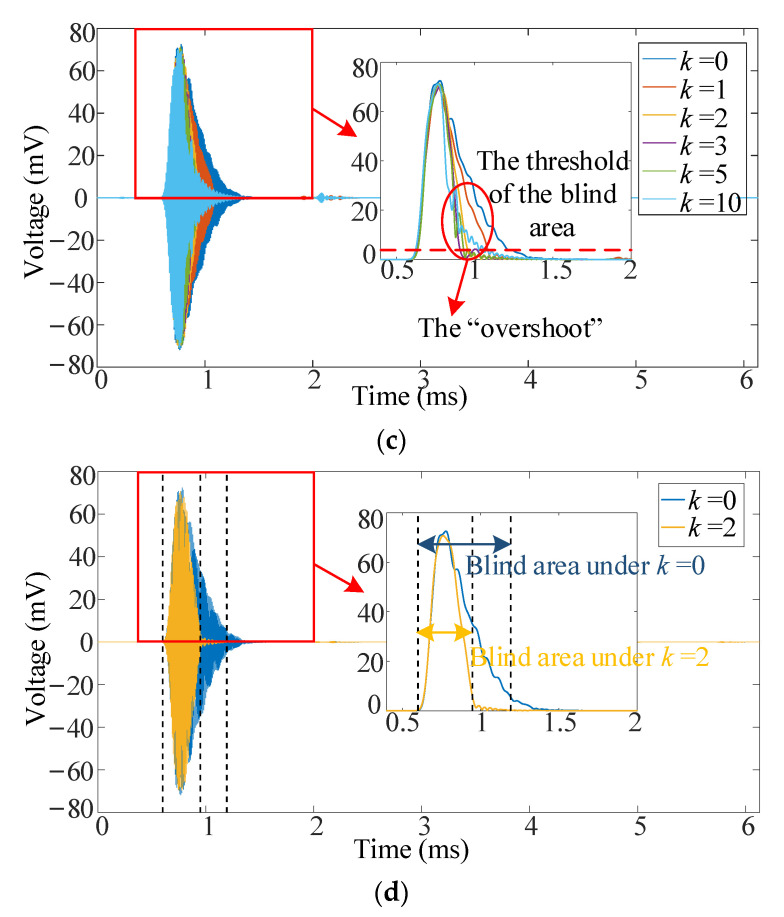
The experimental result of the 115-kHz PMUT array under different *k*: (**a**) the mixed input signal, (**b**) the measurement result of LDV, (**c**) the measurement result of microphone, and (**d**) the measurement result of microphone under *k* = 2.

The suppression signal of ring-down vibration is calculated through MATLAB/Simulink under different proportional parameters (*k*) set as 0, 1, 2, 3, 5, 10, respectively (*k* = 0/1/2/3/5/10). When *k* = 1, the amplitude of the suppression signal is close to the value of the excitation signal. When *k* = 0, the situation is without the suppression system. The input excitation signal is also a 115-kHz sine signal of 20 cycles with an amplitude of 1V_pp_. The processing time at different *k* values in MATLAB/Simulink is about 0.5s. The mixed input signal under different proportional parameters is illustrated in Figure 8a. The amplitude of the suppression signal increases as the proportional parameter.

The vibration displacement of the PMUT array under different proportional parameters is measured through LDV, as shown in Figure 8b. The LDV measurements are also done at the central point of the central element in the PMUT array. The ring-down vibration is reduced significantly by the proposed suppression system, and the attenuation speed of ring-down vibration is faster when the proportional parameter is greater. As shown in Figure 8b, the maximum displacements are almost the same under the same excitation signal, though *k* are different. This proves that the suppression system has no effect on the sensitivity of the PMUT array. The ring-down time of PMUTs is defined as the time corresponding to 37% of the maximum displacement [16]. The ring-down time under different proportional parameters is calculated, as shown in Table 3. The ring-down time is reduced by up to 93% compared to the results without the suppression system. The “overshoot” refers to a phenomenon that the displacement envelope of PMUTs fluctuates around 0 for a period during the ring-down stage. It also can be seen from the measurement results that the central element in the PMUT array is “overshoot” during the ring-down stage when *k* > 2. As the discussion in Section 4.2., the linear transfer function cannot describe the nonlinear influence caused by the mutual influence between the elements of the PMUT array. The nonlinear influence results in an error between the ring-down vibration of the actual PMUT array and that predicted by the transfer function. This error has a great impact on the “overshoot” when *k* > 2. As shown in Figure 8b, the amplitudes of “overshoot” are below the threshold of the ring-down time when *k* > 2. Thus the ring-down time and the effectiveness of the proposed suppression system are not affected by the “overshoot”.

Due to the limitation of LDV, the microphone experiment is conducted to verify the performances of the suppression system in a typical input excitation signal on the PMUT array. The results are illustrated in Figure 8c. The input excitation signal of the microphone experiment is a 115-kHz sine signal of 20 cycles with an amplitude of 5V_pp_. Since the ring-down vibration is suppressed, the blind area of the PMUT array is reduced. The results show that the proposed method is stable between the elements of the PMUT array and is effective for the whole PMUT array. In the short-distance ranging, since the distortion caused by ring-down greatly influences ranging accuracy, the threshold of the blind area should be as small as possible. Whereas in real applications, the noise of PMUTs receivers may also affect the ranging measurement. Considering the above factors, the threshold of the blind area is set as 5% of the maximum output of PMUTs in this study to ensure a decent and robust ranging coverage. The blind area is calculated under different proportional parameters, as shown in Table 4.

In Figure 8c, the trend with different proportional parameters is similar to Figure 8b, and the PMUT array is also “overshoot” during the ring-down stage when *k* > 2. Although the ring-down time is shorter when the proportional parameter is greater, the more significant proportional parameter will cause a stronger “overshoot,” affecting the blind area. Simultaneously, a larger proportional parameter will result in a larger amplitude of the suppression signal, damaging other circuits. Therefore, the optimal proportional parameter is a value that does not generate “overshoot” and does not generate a larger voltage. Then the optimal proportion parameter is 2 for the 115-kHz 3 × 3 PMUT array. The experimental microphone result of *k* = 2 is illustrated in Figure 8d. For the PMUT array, the blind area is 0.367ms when *k* = 2, and the blind area is 0.612ms when *k* = 0. The blind area with the suppression system is reduced by 40% relative to the blind area without the suppression system when *k* = 2.

## 5. Conclusion and Future Work

In this paper, a novel suppression system based on the transfer function of PMUTs is proposed to reduce the blind area of the PMUT array. The proposed system can suppress the ring-down time then decrease the blind area of the PMUT array effectively in a controlled manner. The ring-down time of a 115-kHz PMUT array is demonstrated to reduce by up to 93%. The optimal proportion parameter is derived as two for this PMUT array. Correspondingly, the blind area of the PMUT array is reduced by about 40% under the optimal condition. Moreover, a low ring-down PMUTs system design guideline is proposed based on the suppression system. Compared with the ultrasonic ranging systems in the prior art, this suppression system is insensitive to system parameters and has no impact on the performances of PMUTs. This approach is practical and straightforward to apply, especially when calculation resources are limited in practical industrial scenarios. Furthermore, this approach will be integrated into ASIC as the next step of work.

## Figures and Tables

**Table 1:** Geometric parameters of the PMUT array.

Material	Top Mo	AlN	Bottom Mo	Si	SiO_2_	Cavity
Radius (μm)	700	-	-	-	-	1000
Thickness (μm)	0.2	1	0.2	5	0.5	400

**Table 2:** The system identification results.

Model	Transfer Function	FED
0-2	$H(s)=\frac{4634}{s^{2}+6529s+4.939\times 10^{11}}$	75.75%
1-2	$H(s)=\frac{0.001842s+4944}{s^{2}+6210s+4.926\times 10^{11}}$	81.61%
2-2	$H(s)=\frac{1.222\times 10^{-9}s^{2}+0.0019s+5587}{s^{2}+6227s+4.925\times 10^{11}}$	81.67%
0-3	$H(s)=\frac{-1.202\times 10^{10}}{s^{3}+2.392\times 10^{6}s^{2}+5.072\times 10^{11}s+1.175\times 10^{18}}$	−24.73%
1-3	$H(s)=\frac{5130s+9.596\times 10^{5}}{s^{3}+2.224\times 10^{5}s^{2}+4.938\times 10^{11}s+1.065\times 10^{17}}$	81.52%
2-3	$H(s)=\frac{0.001872s^{2}+4688s-2.062\times 10^{6}}{s^{3}+8486s^{2}+4.925\times 10^{11}s+1.162\times 10^{15}}$	81.48%
3-3	$H(s)=\frac{-6.229\times 10^{-9}s^{3}+0.001823s^{2}+1581s-4.896\times 10^{6}}{s^{3}+8246s^{2}+4.925\times 10^{11}s+1.062\times 10^{15}}$	81.5%

**Table 3:** The result of the ring-down time under different *k*.

*k*	Experiment (s)	Reduction (dB)	Relative Reduction (%)
0	4.1729 × 10^−4^	0	0
1	1.6769 × 10^−4^	−3.9594	59.8150
2	1.0729 × 10^−4^	−5.8989	74.2894
3	7.8487 × 10^−5^	−7.2564	81.1911
5	4.9687 × 10^−5^	−9.2419	88.0929
10	2.8087 × 10^−5^	−11.7139	93.2692

**Table 4:** The result of the blind area under different *k*.

*k*	Experiment (s)	Reduction (dB)	Relative Reduction (%)
0	6.12 × 10^−4^	0	0
1	5.1 × 10^−4^	−0.7918	16.6667
2	3.67 × 10^−4^	−2.2209	40.0327
3	4.39 × 10^−4^	−1.4429	28.2680
5	4.13 × 10^−4^	−1.7080	32.5163
10	4.39 × 10^−4^	−1.4429	28.2680

## Data Availability

Not applicable.
